# Surgical repair for persistent truncus arteriosus in neonates and older children

**DOI:** 10.1186/s13019-020-01114-1

**Published:** 2020-05-11

**Authors:** Rawan M. Alamri, Ahmed M. Dohain, Amr A. Arafat, Ahmed F. Elmahrouk, Abdullah H. Ghunaim, Ahmed A. Elassal, Ahmed A. Jamjoom, Osman O. Al-Radi

**Affiliations:** 1 Cardiac Surgery Division, Department of Surgery, King Abdulaziz University, Jeddah, Saudi Arabia; 2 Pediatric Cardiology Division, Department of Pediatrics, King Abdulaziz University, Jeddah, Saudi Arabia; 3 Pediatric Cardiology Division, Department of Pediatrics, Cairo University, Giza, Egypt; 4 Department of Cardiothoracic Surgery, Tanta University, Tanta, Egypt; 5 Department of Cardiothoracic Surgery, King Faisal Specialist Hospital and Research Centre, Jeddah, Saudi Arabia; 6 Department of Cardiothoracic Surgery, Zagazig University, Zagazig, Egypt

**Keywords:** Persistent truncus arteriosus, Surgical outcome, Late presentation

## Abstract

**Objectives:**

Persistent truncus arteriosus represents less than 3% of all congenital heart defects. We aim to analyze mid-term outcomes after primary Truncus arteriosus repair at different ages and to identify the risk factors contributing to mortality and the need for intervention after surgical repair.

**Methods:**

This retrospective cohort study included 36 children, underwent repair of Truncus arteriosus in the period from January 2011 to December 2018 in two institutions. We recorded the clinical and echocardiographic data for the patients preoperatively, early postoperative, 6 months postoperative, then every year until their last documented follow-up appointment.

**Results:**

Thirty-six patients had truncus arteriosus repair during the study period. Thirty-one patients had open sternum post-repair, and two patients required extracorporeal membrane oxygenation. Bleeding occurred in 15 patients (41.67%), and operative mortality occurred in 5 patients (14.7%). Patients with truncus arteriosus type 2 (*p* = 0.008) and 3 (*p* = 0.001) and who were ventilated preoperatively (*p* < 0.001) had a longer hospital stay. Surgical re-intervention was required in 8 patients (22.86%), and 11 patients (30.56%) had catheter-based reintervention. Freedom from reintervention was 86% at 1 year, 75% at 2 years and 65% at 3 years. Survival at 1 year was 81% and at 3 years was 76%. High postoperative inotropic score predicted mortality (*p* = 0.013).

**Conclusion:**

Repair of the truncus arteriosus can be performed safely with low morbidity and mortality, both in neonates, infants, and older children. Re-intervention is common, preferably through a transcatheter approach.

## Introduction

Truncus arteriosus (TA) represents less than 3% of all congenital heart defects. It is characterized by a common arterial trunk that arises from the base of the heart and supplies systemic, coronary, and pulmonary circulation.1,2 It is associated with ventricular septal defect (VSD) and sometimes, other cardiac lesions such as interrupted aortic arch (IAA), truncal valve stenosis (or regurgitation), and/or hypoplasia of the pulmonary artery branches [1–3].

The treatment of choice of TA, during the neonatal period, in the current era is primary repair [4, 5]. This involves separating the pulmonary and systemic pathways through the establishment of a right ventricle to pulmonary artery connection along with VSD closure [6].

Previous studies have identified IAA and moderate to severe truncal valve regurgitation to be risk factors influencing the mortality after TA repair [1, 6–9].

The Society of Thoracic Surgeons Congenital Heart Surgery Database reported surgical mortality of 9.2% in children and 10.8% in neonates after TA repair [10, 11]. The mortality beyond surgical hospitalization was reported to range from 2 to 15%, according to single-center studies that followed patients for 2–24 years after surgery [2, 7, 8, 12].

Almost all right ventricle to pulmonary artery (RV-PA) conduits require interventions later in life, and it is considered to be the main source of long-term morbidity [12, 13].

Currently, there is no consensus on the optimal conduit that can be used for TA repair [13]. Late presentation of children with TA may make surgery more complicated or even contraindicated due to elevated pulmonary vascular resistance (PVR) [14]. Without surgical intervention, 80% of these patients die within the first year of life, mainly during early infancy [5, 8, 15].

We aim to analyze mid-term outcomes after primary TA repair at different ages and to identify the risk factors contributing to mortality and the need for intervention after surgical repair.

## Patients and methods

### Study population

We retrospectively reviewed the data for all patients who underwent surgical repair of TA from January 2011 to December 2018 at King Abdulaziz University Hospital (KAUH) and King Faisal Specialized Hospital, Jeddah, Saudi Arabia. Approval of the study was obtained from the Institutional Research Ethical Board, and the requirement for individual consent was waived for this retrospective observational study.

### Definitions and clinical data collection

The modified Collett and Edward classification was used to categorizing patients into three subtypes: In type I, the main pulmonary artery (PA) arises from anterior/lateral aspect of the arterial trunk and then branches into the left and right pulmonary arteries. In type II, PA branches originate separate but adjacent from the posterior/lateral aspect of the trunk. In type III, PA branches arise independently from the sides of the common trunk. We excluded the rare type IV from the study, as it is considered, now, a form of pulmonary atresia [16, 17].

Early mortality was defined as death within 30 days of operation or before hospital discharge. Death after that time was considered as late mortality.

We collected the relevant data associated with the initial surgical repair, including demographics, baseline characteristics, operative, and postoperative variables. The clinical outcome measures were recorded, including postoperative mortality, complications, requirement of extracorporeal membrane oxygenation (ECMO) support, duration of mechanical ventilation, vasoactive inotropic score (VIS), intensive care unit (ICU) stay, hospital stay, reoperation, and cardiac catheterization intervention. Excessive bleeding was defined as 7 mL/kg/h or more for 2 or more consecutive hours in the first 12 post- operative hours, 84 mL/kg or more total for the first 24 postoperative hours, or surgical re-exploration for bleeding or cardiac tamponade physiology in the first 24 postoperative hours. VIS was calculated by the following formula using drug dosage in mcg/kg/min: (dopamine + dobutamine) + (milrinone× 10) + (epinephrine× 100) + (norepinephrine× 100) [16].

Preoperative cardiac catheterization was performed for patients who were presented late to evaluate PA pressure and pulmonary vascular resistance index (PVRi). The operability of those patients was determined with the clinical manifestations, chest radiography, echocardiography, and hemodynamic data obtained from cardiac catheterization. Surgical repair was considered if PVRi was less than 8 Wood units [18].

### Surgical procedure

The surgery was performed on the cardio-pulmonary bypass (CBP) through a median sternotomy for all patients. After disconnecting the pulmonary arteries (PAs) from the common trunk, the infundibulum of the right ventricle (RV) was incised, and the VSD was closed with a pericardial patch or a synthetic (Gore-Tex) patch, using an interrupted suture technique. The aortic wall defect was closed directly or repaired with an autologous pericardial patch or bovine pericardial patch.

Continuity between the RV and PA was established with either a bovine jugular valve conduit or an aortic (or pulmonary) homograft. Both truncal and tricuspid valves were routinely inspected, and the appropriate repair was performed if necessary. Additional procedures as IAA repair were performed before reconstruction of the right ventricular outflow tract (RVOT).

The atrial septum was left open if the intraoperative PA pressure was more than 80% of the systemic pressure before weaning from CPB. Electively, the chest was frequently left open, and delayed sternal closure was performed 2 to 3 days later.

### Echocardiographic evaluation and follow-up

We recorded the clinical and echocardiographic data for the patients preoperatively, early postoperative, 6 months postoperative, then every year until their last documented follow-up appointment.

All patients were examined regularly after hospital discharge by a pediatric cardiologist using two-dimensional echocardiography, pulse Doppler and color flow mapping. The ventricular function, degree of regurgitation/stenosis of truncal and pulmonary valves, and any residual defects were assessed. Pressure gradients across the tricuspid valve, the aorta, the conduit, and PAs were also measured. RV pressure of more than 70% of systematic pressure represented an indication for re-intervention.

### Statistical analysis

Continuous data were presented as mean and standard deviation and categorical data as number and percent. Negative binomial regression was used to identify factors affecting the length of hospital stay. Kaplan and Meier’s curve was used to describe the survival distribution and freedom from reoperation. Variables affecting reoperation and mortality were tested using multivariable Cox regression analysis with proportional assumption tested using Schoenfeld residual method. Statistical analysis was performed using Stata 14.2 (Stata Corp, College Town, Texas, USA).

## Results

### Preoperative and operative data

Thirty-six patients had truncus arteriosus repair during the study period. There were 21 male patients (58.33%). Twenty-five patients had type I truncus (69.44%), 7 patients had type II (19.44%), 4 patients had type III (11.11%). Aortic homograft was used in 2 patients (5.56%) and Contegra in 34 patients (94.44%). Deep hypothermic circulatory arrest (DHCA) was used in one patient for the repair of the interrupted aortic arch. (Table 1).

**Table 1:** Preoperative and operative data. (Continuous variables are presented as mean and standard deviation and categorical data as number and percent)

Variables	*N* = 36
**Male**	21 (58.33%)
**Age (days)**	188.5 ± 479.567
**Weight (Kg)**	4.612 ± 4.395
**Height (cm)**	54.676 ± 14.068
**BSA**	0.265 ± 0.152
**Associated anomalies**	
ASD	2 (5.56%)
Interrupted aortic arch	1 (2.78%)
Hypoplastic pulmonary arteries	1 (2.78%)
Pulmonary artery stenosis	1 (2.78%)
DORV	1 (2.78%)
**Previous intracardiac repair**	3 (8.33%)
**Preop pulmonary hypertensive crises**	10 (27.78%)
**Preoperative ventilation**	8 (22.22%)
**Syndromes**	
Di George’s	8 (22.86%)
CHARGE	1 (2.86%)
Adams-Oliver	1 (2.86%)
**Type of the truncus**	
Type 1	25 (69.44%)
Type 2	7 (19.44%)
Type 3	3 (8.33%)
Type 4	1 (2.78%)
**Preoperative truncal valve regurgitation**	
Mild	14 (38.89%)
Moderate	6 (16.67%)
Severe	2 (5.56%)
**Prenatal diagnosis**	2 (5.56%)
**CPB time (min)**	104.059 ± 29.435
**Ischemic time (min)**	78.394 ± 23.285
**Conduit type**	
Aorta homograft	2 (5.56%)
Contegra	34 (94.44%)

*ASD* atrial septal defect, *BSA* body surface area, *CPB* cardiopulmonary bypass, *DORV* double outlet right ventricle

Seven patients underwent truncal valve repair during their initial surgery; six of them had truncal valve regurgitation. The degree of regurgitation was severe in two patients and moderate in five. The seventh patient had moderate truncal valve stenosis and moderate regurgitation. All repairs were successful, and follow up echocardiography showed competent arterio-ventricular valves, and no reintervention was required later for valve-related problems.

### Early operative outcomes

Thirty-one patients had open sternum post-repair, and two patients required extracorporeal membrane oxygenation (ECMO). Bleeding occurred in 15 patients (41.67%), and operative mortality occurred in 5 patients (14.7%). Postoperative outcomes are presented in Table 2. Patients with truncus arteriosus type 2 and 3 had a longer hospital stay. (Table 3).

**Table 2:** Postoperative outcomes. (Continuous variables are presented as mean and standard deviation and categorical data as number and percent)

Variables	*N* = 34
**Postoperative length of stay (days)**	28.727 ± 23.753
**Operative mortality**	5 (14.7%)
**Stroke**	1 (2.78%)
**Cardiopulmonary arrest**	7 (19.44%)
**Complete heart block**	1 (2.78%)
**Bleeding**	15 (41.67%)
**Pneumonia**	10 (27.78%)
**Post discharge mortality**	2 (5.71%)
**Cardiac re-hospitalization**	8 (22.87%)
**ECMO**	2 (5.56%)
**Open sternum**	31 (86.11%)
**ICU stay**	24.029 ± 21.936
**Intracranial hemorrhage**	1 (2.78%)
**Mechanical ventilation (days)**	7.59 ± 6.359
**Inotropic score**	16.35 ± 8.98

*ECMO* extracorporeal membrane oxygenation, *ICU* intensive care unit

**Table 3:** Negative binomial regression for factors affecting the length of hospital stay

Hospital stay	Coef.	*p*-value	95% CI
**Age**	0.0002	0.458	−0.0006- 0.0003
**Gender**	−0.009	0.955	−0.336- 0.317
**Weight**	0.003	0.915	−0.046- 0.0514
**Syndrome**	0.190	0.326	−0.190- 0.570
**Type of truncus arteriosus**			
**2 vs. 1**	0.526	0.008	0.135–0.918
**3 vs. 1**	1.152	0.001	0.499–1.806
**4 vs. 1**	−0.212	0.636	−1.09- 0.666
**Pulmonary hypertension crises**	−0.131	0.483	−0.498- 0.235
**Preoperative ventilation**	1.197	< 0.001	0.844–1.55

### Reoperation and survival

**Fig. 1:**
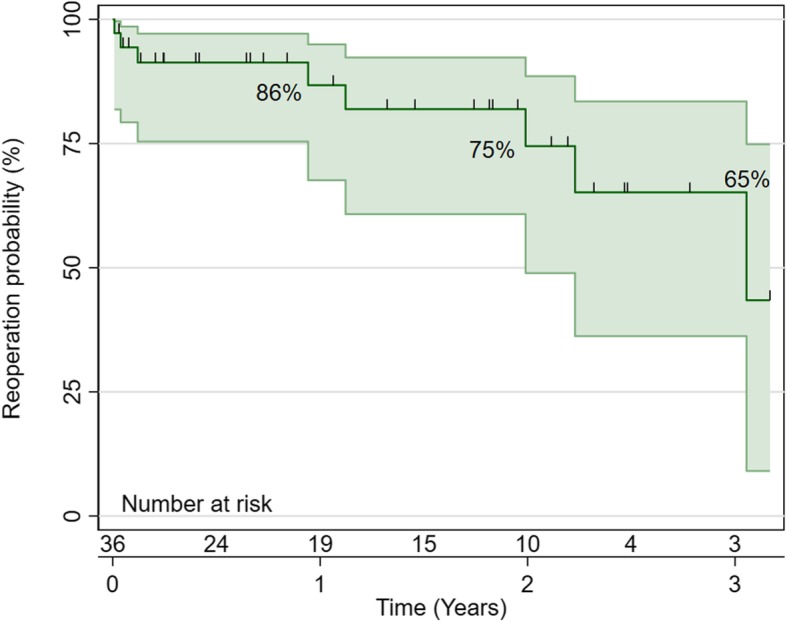
Reoperation free survival, freedom from reintervention was 86% at 1 year, 75% at 2 years and 65% at 3 years

Surgical intervention was required in 8 patients (22.86%), and 11 patients (30.56%) had catheter-based reintervention. (Table 4) Freedom from reintervention was 86% at 1 year, 75% at 2 years and 65% at 3 years. (Fig. 1).

**Table 4:** Re-intervention. (Continuous variables are presented as mean and standard deviation and categorical data as number and percent)

**Reoperation**	8 (22.86%)
Diaphragmatic plication	1 (12.5%)
Redo RV-PA conduit replacement and PA plasty	4 (37.50%)
Re-exploration	2 (25%)
RV-PA conduit	1 (12.5%)
Pulmonary artery plasty	1 (12.5%)
**Catheter-based reintervention**	11 (30.56%)
Diagnostic	2 (18.18%)
VSD closure	1 (9.09%)
ASD closure	1 (9.09%)
Balloon dilatation of the LPA and RPA	1 (9.09%)
RPA stenting	1 (9.09%)
Bilateral PA balloon dilatation + stenting	2 (18.18%)
LPA stenting	1 (9.09%)
Balloon dilatation of the Left main bronchus	1 (9.09%)
MPA-LPA junction stenting	1 (9.09%)
PA stenting	1 (9.09%)

*ASD* atrial septal defect, *LPA* left pulmonary artery, *MPA* main pulmonary artery, *PA* pulmonary artery, *RPA* right pulmonary artery, *RV* right ventricle, *VSD* ventricular septal defect

**Fig. 2:**
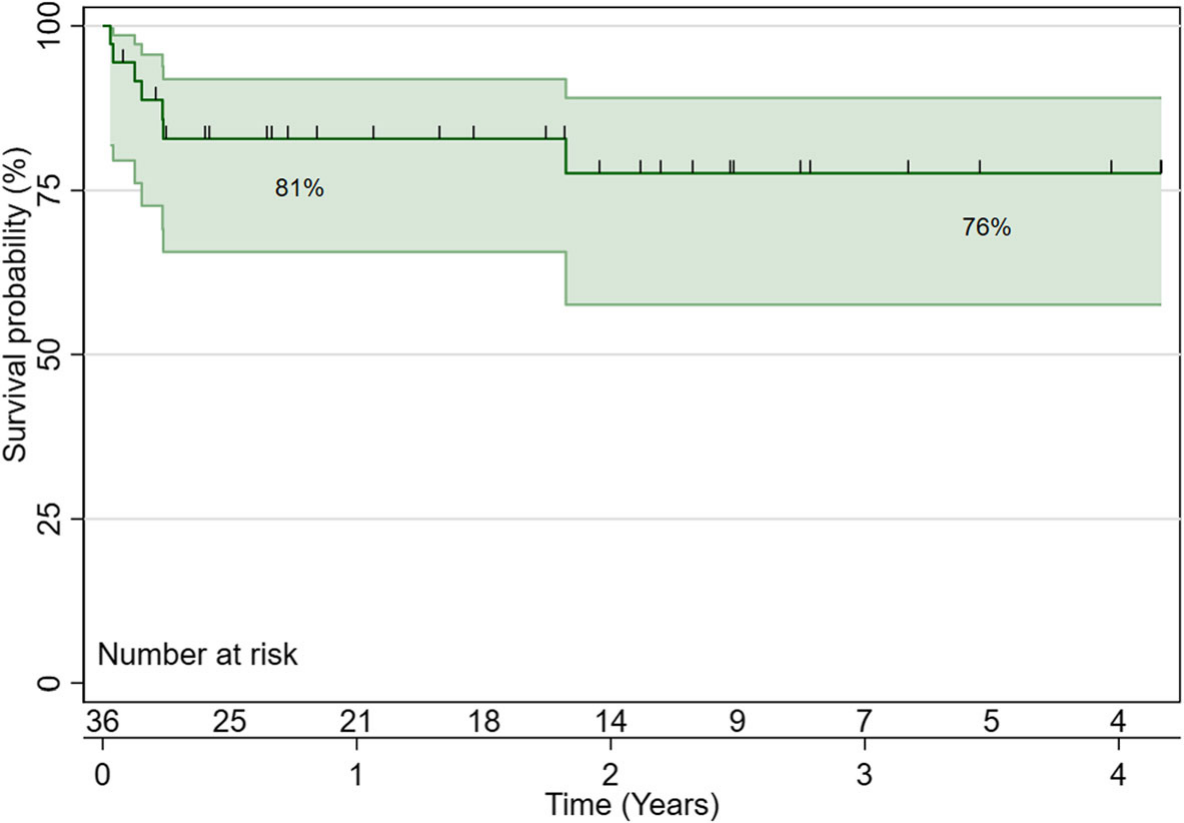
Kaplan-Meier survival distribution Survival at 1 year was 81% and at 3 years was 76%

We reported seven deaths, five of them were in the hospital. The causes of death were a septic shock in three cases, and two cases had a cardiac arrest, and ECMO was instituted as a part of ECPR, both patients did not survive to discharge due to multi-organ failure. We reported two late mortalities, and the cause of death for those patients was not defined. Survival at 1 year was 81% and at 3 years was 76%. (Fig. 2).

High postoperative inotropic score predicted mortality; however, none of the preoperative and operative variables predicted the need for reoperation. (Table 5).

**Table 5:** Multivariable Cox regression for factors affecting reoperation and mortality

Reoperation	HR	*p*-value	95% CI
**Age**	0.999	0.763	0.992–1.006
**Gender**	0.316	0.222	0.05–2.004
**Weight**	0.858	0.618	0.471–1.564
**Syndrome**	0.849	0.855	0.147–4.922
**Truncal valve regurgitation**	0.321	0.206	0.055–1.87
**Truncal valve stenosis**	0.772	0.342	0.452–1.318
**Type of truncus arteriosus**	1.550	0.433	0.518–4.637
**Pulmonary artery plasty**	0.312	0.234	0.046–2.126
**Mortality**			
**Age**	1.004	0.290	0.996–1.013
**Weight**	0.936	0.772	0.598–1.467
**Truncus arteriosus type**	10.393	0.210	0.267–404.788
**Associated syndromes**	0.413	0.544	0.023–7.192
**Truncal valve regurgitation**	1.364	0.641	0.37–5.032
**Truncal valve stenosis**	0.885	0.800	0.34–2.266
**Postoperative VIS**	1.137	0.013	1.028–1.259

## Discussion

Complete one stage repair is the treatment of choice for TA and should be performed early within the first few months of life.1 Staged repair of TA was described in association with other anomalies [16]. All our patients had single-stage repair with the repair of concomitant anomalies; one patient underwent DHCA for the concomitant repair of the interrupted aortic arch.

Operative mortality was reported in 5 patients (14.7%), and survival at one-year was 81%. Tlaskal and associates reported 23% early mortality, and Schrieber and colleagues had 21% early mortality, which improved to 13% in the recent era [1, 17]. The highest mortality after TA repair was reported to occur in the first-year post-repair. Rajasinghe and associates [18] reported 56% of deaths that occur after TA were in the first year, and Brizard and colleagues [19] had 87% of deaths that occurred in the first-year post-repair.

Several risk factors were associated with mortality. Several studies demonstrated the association between mortality and IAA [19, 20]. In our series, one patient had IAA and was discharged after the successful repair of TA and the associated IAA. In other series, ECMO was a risk factor for operative mortality [21]. In this study, two patients had ECMO, and both of them died [22, 23]. Naimo and colleagues [24] found that 32% of DiGeorge syndrome had operative mortality; similarly, in our study, 25% of DiGeorge syndrome had operative mortality. All patients who had mortality were males.

Reoperation is common after TA repair, and the freedom from the reoperation in our series was 65% at 3 years. Naimo and his group in their 35 years’ experience at Melbourne University, reported freedom from reoperation was 23% at 10 years [24]. The same group from Melbourne found that reoperations after TA repair were not related to the preoperative truncal valve regurgitation [25]. None of our patients had reoperation for the truncal valve lesion. In other series, the freedom from reoperation for the truncal valve was 83.9% at 15 years [26].

We had 19 reinterventions, either surgical or catheter-based most of them were related to pulmonary artery stenosis. Similar to other series, which reported reoperation for RVOT obstruction and arch obstruction [26, 27]. Several techniques were described to decrease the RVOT reoperation [28]. IAA was associated with a high reoperation rate because of arch obstruction; we had one patient with IAA who did not require further reoperation.

### Study limitations

The major limitation of the study is the retrospective nature; however, it is an accepted study design for this rare anomaly. The number of patients is relatively small patients’ number with a small number of events.

## Conclusion

Repair of the truncus arteriosus can be performed safely with low morbidity and mortality, both in neonates, infants, and older children. Re-intervention is common, preferably through a transcatheter approach.

## Data Availability

General data are part of the Congenital Cardiac Surgery Database (CCSdb) available at www.ccsdb.org and were populated prospectively. Specific data and materials are available with the corresponding author.

## Abbreviations

TA: Truncus arteriosus
VSD: Ventricular septal defect
IAA: Interrupted aortic arch
(RV-PA): Right ventricle to pulmonary artery
PVR: Pulmonary vascular resistance
PA: Pulmonary artery
ECMO: Extracorporeal membrane oxygenation
VIS: Vasoactive inotropic score
ICU: Intensive Care Unit
PVRi: Pulmonary vascular resistance index
CPB: Cardiopulmonary Bypass
RV: Right ventricle
RVOT: Right Ventricular Outflow Tract
DHCA: Deep hypothermic circulatory arrest
